# Dabigatran: A new oral anticoagulant. Guidelines to follow in oral surgery procedures. A systematic review of the literature

**DOI:** 10.4317/medoral.21202

**Published:** 2016-10-01

**Authors:** Marta Muñoz-Corcuera, Lucía Ramírez-Martínez-Acitores, Rosa Mª López-Pintor, Elisabeth Casañas-Gil, Gonzalo Hernández-Vallejo

**Affiliations:** 1 DDS, PhD. Assistant professor. Oral medicine specialist, Complutense University, Madrid, Spain; 2 DDS. Assistant professor. Oral medicine specialist, Complutense University, Madrid, Spain; 3 DDS, PhD. Associate professor. Department of Oral medicine and Orofacial surgery. Faculty of Odontology. Complutense University, Madrid, Spain; 4 DDS, PhD Student. Assistant professor. Oral medicine specialist. Complutense University, Madrid, Spain; 5 MD, DDS, PhD. Professor. Department of Oral Medicine and Orofacial Surgery. Faculty of Odontology. Complutense Univer-sity. Madrid, Spain

## Abstract

**Background:**

Dabigatran is a newly commercialized drug that is replacing other anticoagulants in the prevention of venous thromboembolism, stroke and systemic arterial valve embolism. It acts directly on thrombin presenting in a dynamic and predictable way, which does not require monitoring these patients. Therefore, we consider the need to assess whether their use increases the risk of bleeding involved before any dental treatment.

**Material and Methods:**

We performed a systematic review with a bibliographic search in PubMed/Medline along with the Cochrane Library. We excluded articles dealing with all anticoagulants other than dabigatran, and works about surgical treatments in anatomical locations other than the oral cavity.

**Results:**

We included a total of 13 papers of which 1 was a randomized clinical trial, 9 narrative literature reviews, 1 case series, 2 clinical cases and 1 expert opinion. Because we did not obtain any properly designed clinical trials, we were unable to conduct a meta-analysis.

**Conclusions:**

Currently, there is no consensus on the procedure to be followed in patients taking dabigatran. However, all authors agree to treat each case individually in accordance to the risk of embolism, postoperative bleeding and renal function. Also, it is necessary to perform minimally invasive interventions, and take the appropriate local anti-hemolytic measures.

**Key words:**Oral anticoagulants, dabigatran, risk of bleeding, oral surgery, dentistry.

## Introduction

Atrial fibrillation is the most common cardiac arrhythmia and a major cause of stroke in the United States and Europe. There is an estimated 2.2 million people suffering from this disease in the United States, and 4.5 million in Europe. In patients with atrial fibrillation, 80% of heart attacks cause death or disability, and mortality per year reaches 50%. Vitamin K antagonists such as warfarin and acenocoumarol are very effective in preventing strokes in patients with atrial fibrillation, and have been for many years; thus being the only drugs available for long-term anticoagulant therapy (1,2).

**Figure 1:**
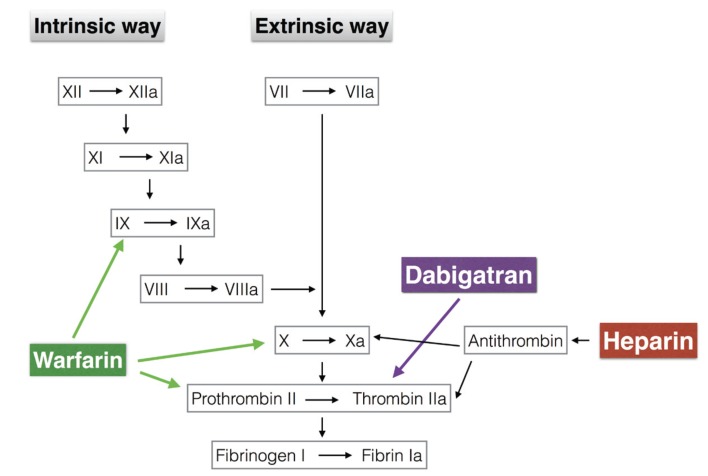
Action levels of heparin, warfarin and dabigatran in the intrinsic and extrinsic coagulation pathways.

Warfarin and acenocoumarol exert its anticoagulant effects by reducing the levels of prothrombin and factor X; whereas heparin acts by binding to antithrombin and enhancing its ability to inhibit thrombin (Fig. 1). With regards to heparin, its main disadvantage is that it causes an indirect inhibition of thrombin unpredictable, as it is dependent on the availability of antithrombin. On the other hand, warfarin has its drawbacks with a narrow therapeutic window (the range in which a drug can be used without causing toxic or lethal effects on a living organism), the need to monitor the state of anticoagulation of a patient by controlling the International Normalized Ratio (INR), the numerous food and drug interactions, lack of direct action on coagulation proteins and high start activity time and its removal (3-5). This large amount of disadvantages has been the basis for the development of new oral anticoagulants, which act directly inhibiting thrombin (1-6).

Dabigatran etexilate (Pradaxa, Boehringer Ingelheim Pharmaceuticals Inc., Ridgefield, CT), is a direct thrombin inhibitor, which was approved by the European Medicines Agency in 2008, and the Food and Drug Administration (FDA) in October 2010 for the prevention of stroke and systemic embolism in patients with non-valvular atrial fibrillation, as well as, for the prevention of venous thromboembolism after orthopedic surgery for total hip replacement or knee (4).

However, patients taking warfarin or acenocoumarol have a pattern of reduction of the drug and a reference INR values that can be followed without the patient´s risk of bleeding during dental procedures; yet there are no guidelines for managing patients taking dabigatran before dental procedures that can cause bleeding.

The aim of this paper is to perform a systematic review and meta-analysis summarizing if the use of dabigatran increases the risk of bleeding before dental procedures that involve bleeding, and if that risk is greater than that produced by conventional anticoagulants. Later, we will try to give a protocol of dental procedures that involve bleeding in patients treated with dabigatran.

## Material and Methods

- Defining the questions:

To carry out this systematic review the questions we asked ourselves were as follows:

Does the use of dabigatran increases the risk of bleeding when performing dental treatments which involve bleeding?; Is the risk of bleeding before such dental procedures involving bleeding greater than that produced by classic anticoagulant treatments such as heparin and warfarin?, and are there protocols for handling patients who take dabigatran prior to dental procedures that involve bleeding?

- Search strategy:

To carry out this systematic review we conducted a literature search in PubMed / Medline, and Cochrane Library databases using key words “dabigatran” AND “dentistry” and “dabigatran” AND “oral surgery”. Only papers with human subjects were selected. The titles and abstracts that resulted from the search were reviewed, and full-texts papers that were considered relevant to the review were read. Two authors (MMC and LRM) reviewed the papers selected independently. We compared the lists, and when in disagreement, underwent discussion based on the inclusion and exclusion criteria. We then sought manually for additional papers reviewing the references on selected articles.

- Inclusion and exclusion criteria:

Inclusion criteria.

All articles published in English, that discuss the risk of bleeding from dabigatran after dental treatment, were included. We included narrative reviews, case reports, case series, case-control studies, prospective studies, randomized clinical trials, and systematic reviews. We searched articles published in the last 10 years. The last electronic search was conducted in 15th October 2015. We selected only studies conducted in adult patients.

Exclusion criteria:

Studies were excluded if they were published in a language other than English. Papers that assessed the bleeding caused by other anticoagulants (apart dabigatran) were rejected. We did not include studies realized in animals.

- Quality assessment

In the final selection of eligible studies, we assessed features that could potentially bias following the recommendations by Cochrane for assesing risk of bias. Critical appraisal was conducted by two reviewers (MMC and LRM) independently of each other. The reviewers met to discuss the results of their critical appraisal, if the two reviewers disagreed on the final critical appraisal and could not be resolved through discussion, a third reviewer (RLP) was required.

## Results

**Figure 2:**
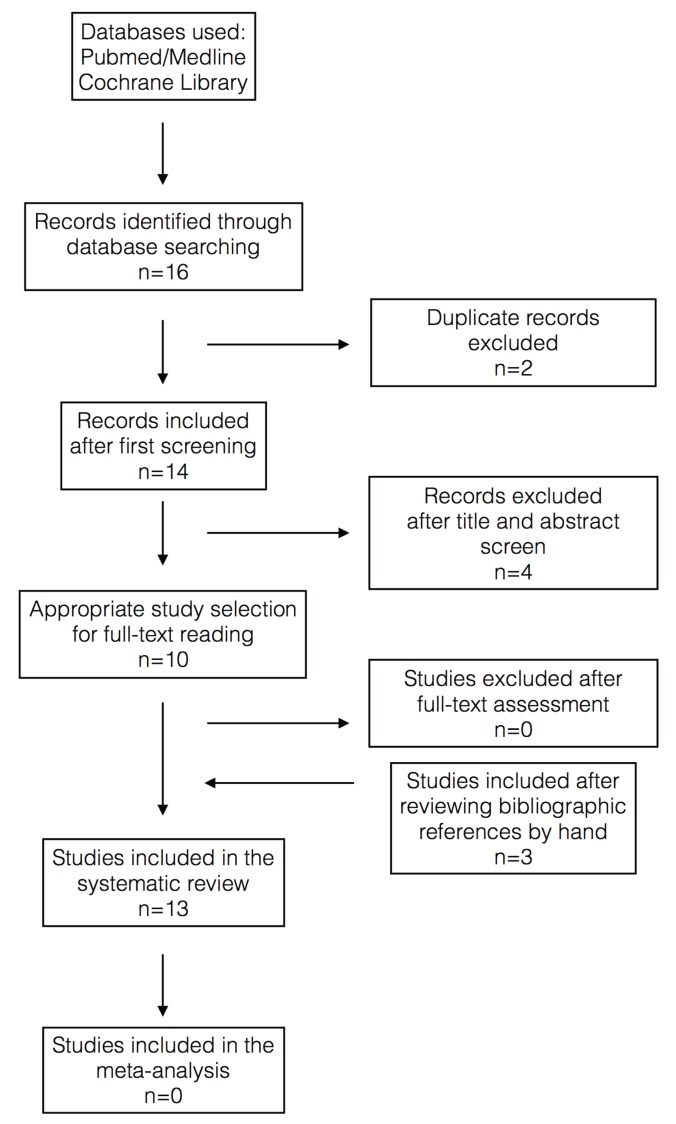
Search strategy and selection diagram.

In figure 2, the search strategy and article selection is discussed. A total of 13 papers were included, 1 randomized clinical trial, 9 narrative literature reviews, 1 case series, 2 clinical cases, and 1 expert opinion ( Table 1).

**Table 1:**
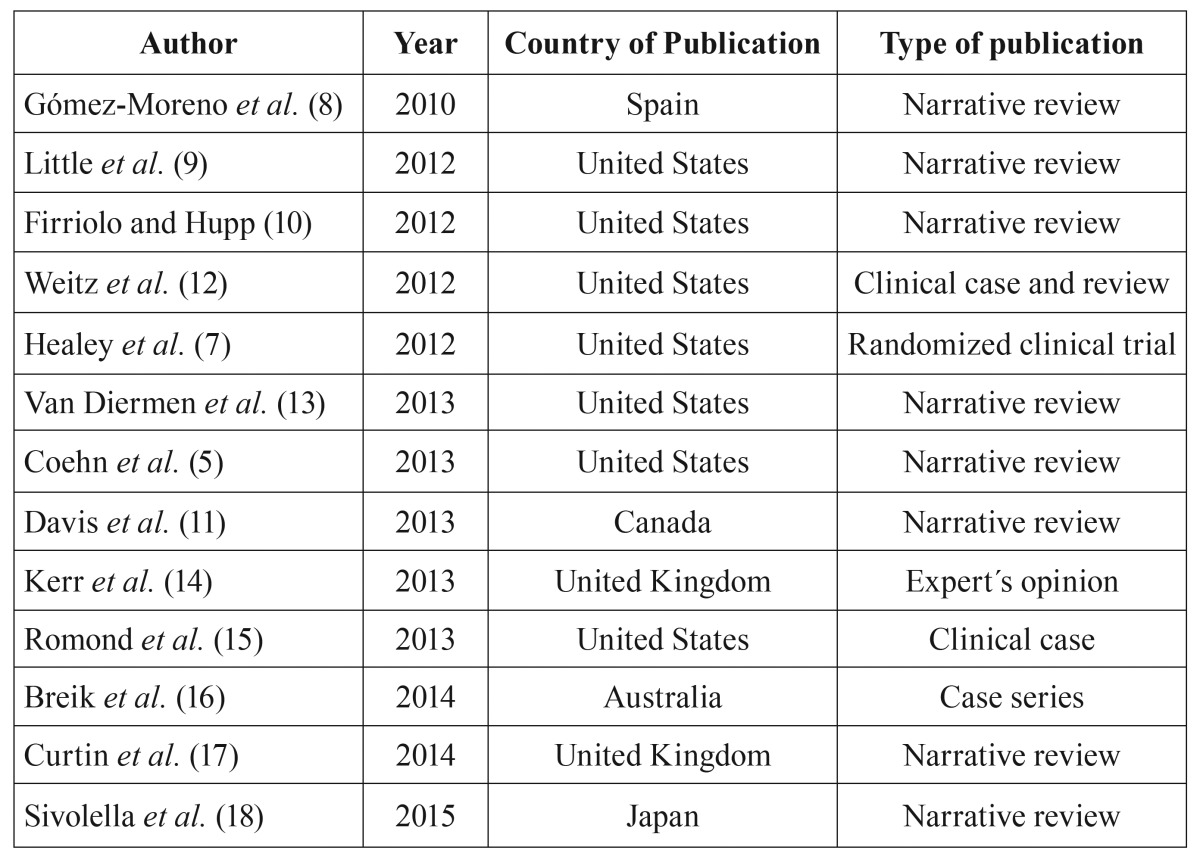
Papers included in the systematic review.

We only found two papers that responded to the question: Does the use of dabigatran increase the risk of bleeding in dental treatments that involve bleeding? The papers selected were two clinical cases, therefore of very poor methodological quality. Regarding the answer to the question: Is the risk of bleeding before dental procedures that involve bleeding in patients taking dabigatran greater than that produced by classical anticoagulants (heparin and warfarin)? We could not find any papers that specifically answers that question; the study by Healy (7) in 2012 includes dental procedures among those evaluated, but does not specify subsequent bleeding complications specific to the type of surgery that the patients underwent. And yes, there are studies that answer the question, are there protocols for handling patients (taking dabigatran) before dental procedures that involve bleeding? 

All the studies reviewed provide different protocols for managing these patients, but none of these papers are based on results of methodological, well-designed studies, just mere opinions and recommendations from commercial brands and / or experts.

Because we could not get properly designed clinical studies, we were unable to do a meta-analysis. We will describe in a summarized manner each of the selected papers.

Gómez-Moreno *et al.* (8) in their review in 2010, mentioned the lack of practical guidelines, as well as a lack of protocol to reduce drug dosage before dental procedures, and the importance of local hemostatic measures. Also, they emphasize among the advantages of dabigatran that it is a drug that does not interfere with antimicrobial antibiotics, most widely used in dentistry nowadays.

In the review by Little *et al.* (9) in 2012, in light of the revised articles, the authors conclude that patients treated with dabigatran may undergo invasive dental procedures without altering the dose of the medication. The dentist should consult the patient’s physician to plan the procedure, and confirm whether the patient will continue with the same dose after surgery. To manage possible bleeding, local measures should be used. In cases requiring extensive oral surgery, it is important to consult with the patient´s physician to determine an action plan, in order to prevent thromboembolism, as well as, excessive bleeding.

Firriolo and Hupp (10), in 2012 and Davis *et al.* (11), in 2013 also published reviews and recommendations based on other authors´ recommendations, as well as, the pharmacological properties of dabigatran; and they noted that it does not seem necessary to remove the drug prior to dental treatment, especially if local and adjuvant hemostatic measures are taken (suture, gelatin or cellulose sponges, tranexamic acid rinses at 4.8% for 2-5 days). However, in cases where excessive bleeding or hemostatic problems are expected, dabigatran should be removed at least 24 hours before surgery or more depending on the risk of bleeding, renal function, and the presence of other conditions they could increase the bleeding ( Table 2). Due to the anticoagulant effect, that quickly establishes, the drug should not be taken immediately after surgery, instead once the clot is stabilized (24-48 hours after surgery).

**Table 2:**
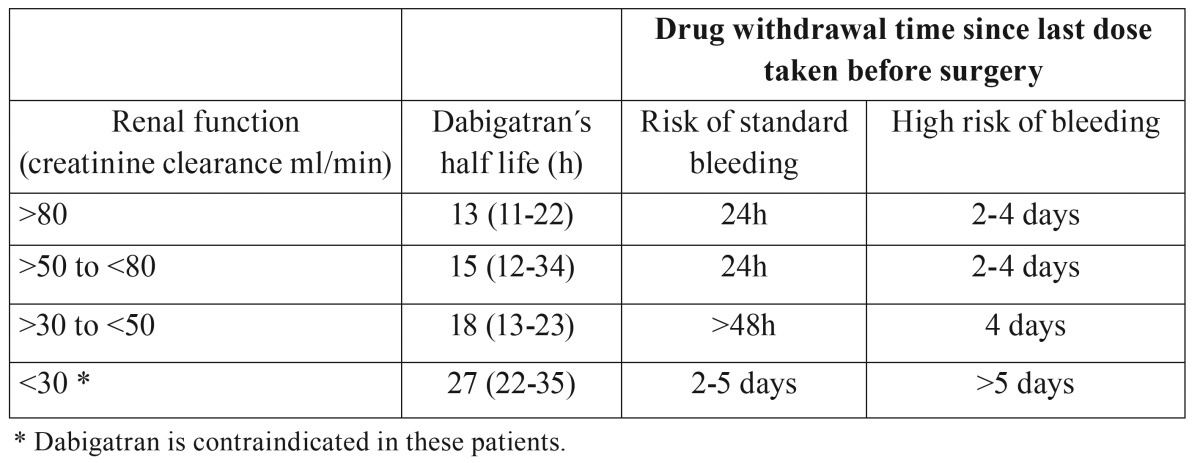
Guide to discontinue use of dabigatran prior to surgical procedures.

Weitz *et al.* (12) in 2012 published a case study of a patient taking dabigatran which presented bleeding complications. In the article, the authors do a systematic reviewed and include recommendations for minor surgical procedures, such as no drug withdrawal in dental cleanings and extractions, and to do such procedures more than 10 hours after taking last dose of the drug.

Van Diermen *et al.* (13) in 2013 conducted a review aimed at finding dental studies regarding the management of anticoagulated patients, including those taking new anticoagulants; and to propose a patient management guide for general practitioners. Regarding dabigatran in patients subjected to simple dental treatments (up to 3 extractions, up to 3 implants, scaling and root planning, flap surgeries, alveoloplasties and apicoectomies), the authors made the following recommendations: To not stop taking dabigatran; Warn patients not to take their medication within 3 hours immediately after surgery; and adopt local, pre and post-operative measures, such as minimizing surgical trauma, suture wounds, the use local hemostatics or apply pressure locally; also providing the patient with written instructions on how the post-operative measures should be, and the steps that the patient should take if he/she experiences bleeding.

Cohen *et al.* (5) in 2013 conducted a review and developed a small clinical management guide; they advised that the correct clinical care at the dental office should begin by writing a thorough medical history of the patient, collecting any previous episodes of excessive bleeding associated with anticoagulants or diseases of any kind. These authors suggest that basic oral surgery procedures such as few tooth extractions or localized periodontal surgery must be done at the first visit to assess the bleeding, followed by local hemostatic measures such as suture or gelatin sponges. If surgery is more complex or major bleeding is expected, one should consider withdrawing the drug for 48 hours, and after consulting with the patient´s physician. If the procedure post-operative healing is good, the patient could continue the medication the day after surgery. All these guidelines relate to a healthy patient without kidney or liver disease; in such cases, the drug should be withdrawn 4-5 days depending on the physician´s suggestions.

Kerr *et al.* (14) published in 2013 a letter in which, as experts, proposed a management guide that suggests to not withdraw the medication, requiring the use of atraumatic extraction techniques, limiting the number of teeth extracted in the same procedure to 3-4, and local hemostatic measures (sutures, local pressure and local hemostatics). With this, they consider the treatment will be safe in a regular dental practice. It is also noted that such patients with recently placed stents, liver or kidney failure, alcohol problems, medicated with cytotoxic drugs and with clotting problems, should have the practitioner consult their physician before performing any dental procedures that cause bleeding.

Romond *et al.* (15) in 2013 published the case report of a patient who had eight dental extractions and pre-prosthetic surgery (alveoloplasty and remodeling of the tuberosity in the maxilla) who was also taking dabigatran. In this case, the patient withdrew dabigatran 24 hours before the procedure, and surgery was performed under intravenous sedation and local hemostatic measures were taken, such as the use of local anesthesia with vasoconstrictor, gelatin sponges, suture and placement of the immediate prosthesis. There was no excessive bleeding or clotting problems in this case, making the healing process correct. The authors note that having no agent to reverse the action of dabigatran is sufficient reason for withdrawing the medication when the procedure is more invasive than 2-3 extractions.

Breik *et al.* (16) in 2014 published a guide of recommendations based on a series of cases of 5 patients who had single and multiple extractions. These authors recommended to not removing the dabigatran in procedures with less risk of bleeding, such as periodontal treatment, dental restorations with matrices, root canals or single tooth extractions. For the control of postoperative bleeding in simple uncomplicated extractions, local measures such as mechanical pressure, sutures and the use of local hemostatics are recommended. In cases where performing several extractions, the patient should be referred to his/her physician to assess the risk of withdrawing dabigatran 24 hours before surgery. If renal function is impaired, the withdrawal is assessed 48 hours earlier. Activated partial thromboplastin time (aPTT) or thrombin time (TT) can be assessed previously to check the status of the patient anticoagulation. After the surgery, local hemostatic measures must take place and dabigatran can be resumed 24-48 hours later.

Curtin *et al.* (17), in their review in 2014 also highlight the absence of clear clinical guidelines and recommend that dentists must know the existence of new anticoagulants, both brand name and generic, consider possible drug interactions and to consult with a patient´s physician before performing invasive treatments which may cause bleeding.

Sivolella *et al.* (18), published in 2015 a review summarizing all prior publications about management of patients taking dabigatran and undergoing oral surgery procedures. They concluded that the management of these patients is essentially based on the average life of the drug and kidney condition of the patient. Furthermore, they note that there are no clear guidelines to manage these patients, and clinical trials are needed to establish the protocols in the future.

 Table 3 summarizes the pre and post-operative recommendations published by all the different authors in this review with regards to dabigatran.

**Table 3:**
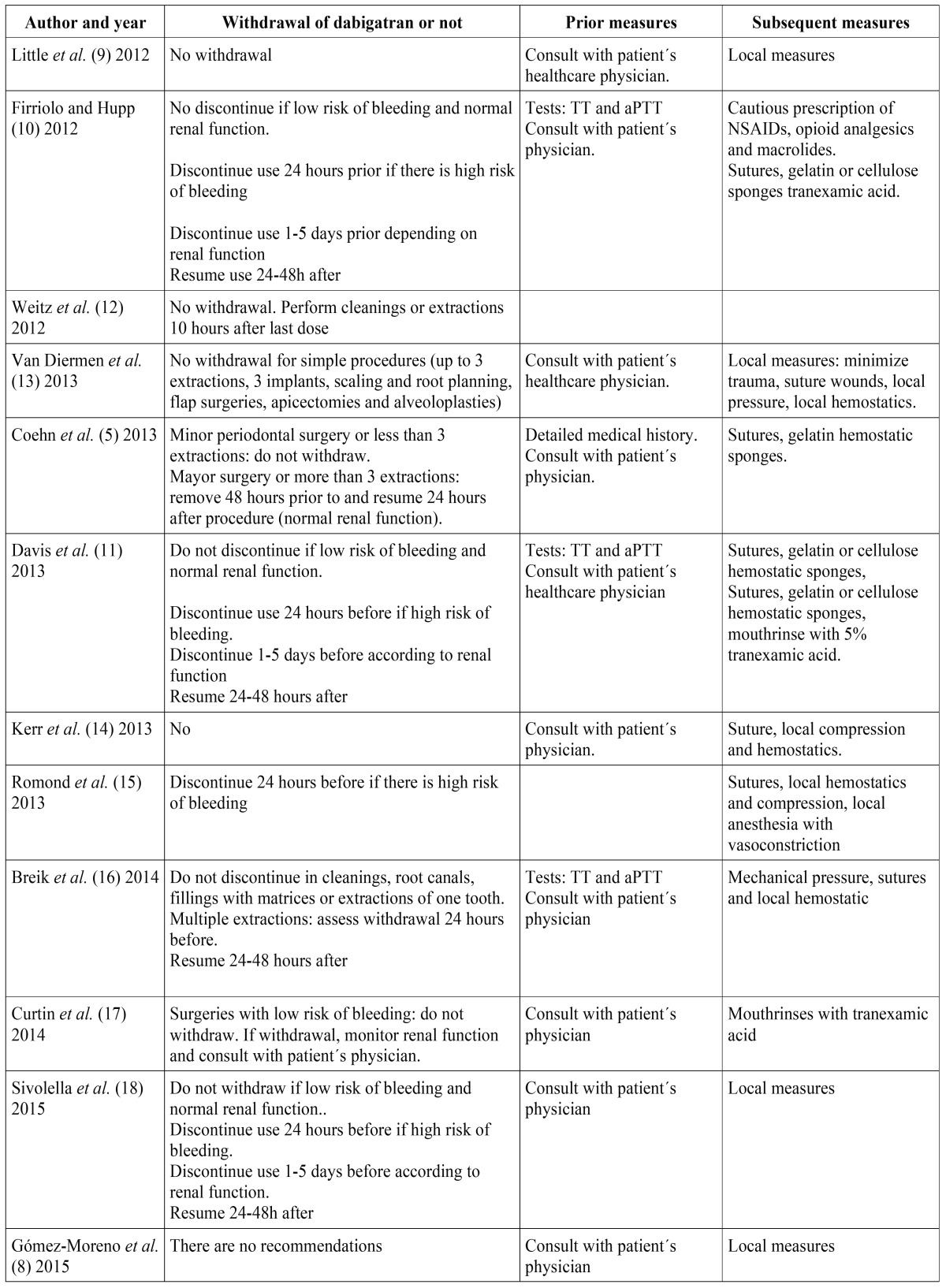
Summary of papers regarding patients taking dabigatran who are undergoing dental procedures that cause bleeding.

## Discussion

Due to the fact that we have not found properly designed clinical studies, we will divide the discussion into two sections. The first section will summarize the drug´s characteristics and how those can be an influence when carrying out dental treatments, and the second part, the management of anticoagulated patients with dabigatran and what to do with procedures that involve bleeding.

- Dabigatran´s characteristics

Dabigatran etexilate produces a dose-dependent prolongation and nonlinear aPTT, while values such as TT, ecarin clotting time (ECT, test using the poison from the Echis carinatus snake) and INR rise in a linear fashion and are dose-dependent. There is a close relationship between plasma concentration of dabigatran, and the prolongation of blood clotting. Dabigatran acts directly on plasma thrombin, since it has been observed that the maximum effect on coagulation parameters occurs simultaneously with the maximum concentration observed in plasma (3,10,13).

Dabigatran is administered as a prodrug, dabigatran etexilate, which has a rapid absorption via gastrointestinal tract, and quickly becomes dabigatran; 20% of the drug is metabolized in the liver, and 80% is excreted primarily by the kidneys; thus having a half-life of about 12 to 17 hours. Patients with a creatinine clearance <50 ml/min may have a longer elimination and higher drug levels in plasma. In patients with severe renal failure (creatinine clearance <30 ml/min), dabigatran should be used with caution or contraindicated (3,4,9,11,15).

Dabigatran´s peak effect occurs two hours after administration of the first dose, reaching a state of stability 3 days after starting treatment with a regular dosage (e.g., 150 mg twice daily). The effect duration is approximately 22 hours.

The advantage of this drug is that it does not need motorization of the patient by means of coagulation tests (INR), thus far, introducing stable and predictable pharmacodynamics 5,9. However, there is no coagulation measurement that could accurately predict the degree of anticoagulation in a patient medicated with dabigatran, and there are no guidelines for routine monitoring of coagulation in these patients (3,5,13).

Moreover, there is no consensus on what lab test is more effective to assess the clotting level in a patient, since measuring INR, a test familiar to physicians, does not display correctly the degree of coagulation of the patient (17,19). The most accessible laboratory tests to determine the presence or absence of anticoagulant effect in patients taking dabigatran in case of emergencies are the aPTT and TT (15,19); however, the most appropriate and sensitive test to quantify the anticoagulant effect of this drug are ECT, dTT (diluted thrombin time) and the trial thrombin inhibitor Hemoclot ® (Hyphen BioMed, Neuville-sur-Oise, France) (10,18,19).

In Erope, dabigatran has been approved for the prevention of venous thromboembolism after orthopedic surgery, such as total hip or knee replacement, and the prevention of stroke and systemic arterial embolism in high-risk patients with non-valvular atrial fibrillation (1,2,11,12,18).

A meta-analysis conducted in 2012 by Miller *et al.* (20) on the efficacy and safety of new anticoagulants (dabigatran, rivaroxaban, apaxiban) versus warfarin in patients with atrial fibrillation, found that new anticoagulants are more effective than warfarin for the prevention of stroke and systemic embolism in patients with atrial fibrillation. Also they noted that intracranial hemorrhage risk is less, and therefore, have a higher safety than warfarin. Nevertheless, Healy *et al.* (7) have determined that dabigatran has an increased risk of bleeding when the patient is subjected to an urgent surgical procedure; in non-urgent cases, the risk of bleeding is similar in both dabigratran and warfarin.

Dabigatran is well tolerated in single and multiple doses without occurrence of serious adverse effects. Minor adverse reactions include headache, gastrointestinal disturbances, peripheral edema, back pain, arthralgia, nasopharyngitis and insomnia (3,11). There is a high incidence of dyspepsia, gastritis-like symptoms, and hypersensitivity reactions (4,9).

Dabigatran has few interactions that are relevant from a clinical point of view. Rifampicin reduces the anticoagulant effect of dabigatran, and as can also do other inducers of P-glycoprotein such as dexamethasone and carbamazepine. Inhibitors of P-glycoprotein as ketoconazole, itraconazole, erythromycin, clarithromycin, verapamil and amiodarone tend to increase the anticoagulant effect. Its use with other anticoagulants, antiplatelet agents, NSAIDs, salicylates and certain herbs (alfalfa, anise...) do not pose an interaction, but they can increase the risk of bleeding, and therefore, should be avoided. Drugs that can be used to control pain in patients taking dabigatran are opioids or acetaminophen (9-11).

In conclusion, we can add that the advantages of dabigatran are speed of action, the wide therapeutic window, the low potential to cause interactions with other drugs or foods, and predictable anticoagulant effect, which eliminates the need for routine patient monitoring and management of the drug in fixed-doses (18).

On the other hand, its disadvantages include the patient in treatment with dabigatran should comply strictly with taking scheduled doses (4,9). In addition, there is a high incidence of dyspepsia, gastritis-like symptoms and hypersensitivity (urticaria, rush, pruritus and anaphylactic shock) (4,8). It has also been seen, when compared with warfarin, the bleeding risk is greater when the patient undergoes a surgical procedure (7). In cases of major bleeding, there is no antidote to reverse the anticoagulant effect (4,9); although some researchers have recently identified a possible antidote, an antibody fragment a Dabi-Fab, which directly neutralizes dabigatran (21).

- Management of patients anticoagulated with dabigatran prior to dental procedures that involve bleeding.

There is no consensus regarding how to manage a patient medicated with dabigatran who needs to undergo a surgical procedure. However, all authors agree that we should individualize each case taking into consideration the risk of bleeding during surgery, along with the risk of embolism (if we discontinue the medication) and renal function of the patient.

Healey *et al.* (7) in 2012 conducted a randomized clinical trial which compared the risk of bleeding after surgical procedures (including oral surgery as well) in patients taking dabigatran and patients taking warfarin, and came to the conclusion that bleeding complications are similar in both cases. Authors like Golembiewski advise that dabigatran should be discontinued before any surgical procedure that could have a bleeding risk. Drug withdrawal time is dependent on such bleeding risk, (standard or high) and renal function of the patient (4).

Unlike what happens with antiplatelet and anticoagulants such as warfarin or acenocoumarol, for which there are clear guidelines for action on withdrawing or not the medication, as well as pre and postoperative measures taken with patients in the dental practice (5,13); nowadays there are recommendations for dabigatran based only on the pharmacological properties of the drug, and on experiences with isolated case reports or small groups of patients.

Almost all of the studies reviewed focus on patient management during extractions, with no papers and no specific recommendations for other oral treatments that produce bleeding, such as dental cleanings or scaling and root planning.

In cases in which minor bleeding occurs after surgery, it is recommended to postpone the next dose of the drug and adopt local measures. If bleeding is severe, treatments available at the hospital level include mechanical compression, surgical interventions, fluid replacement, hemodynamic support, intake of activated carbon and hemodialysis (2).

In view of this systematic review, in which we have found no reliable clinical data derived from clinical trials and general recommendations for the management of these patients in the dental office, we could include the following recommendations ( Table 4):

**Table 4:**
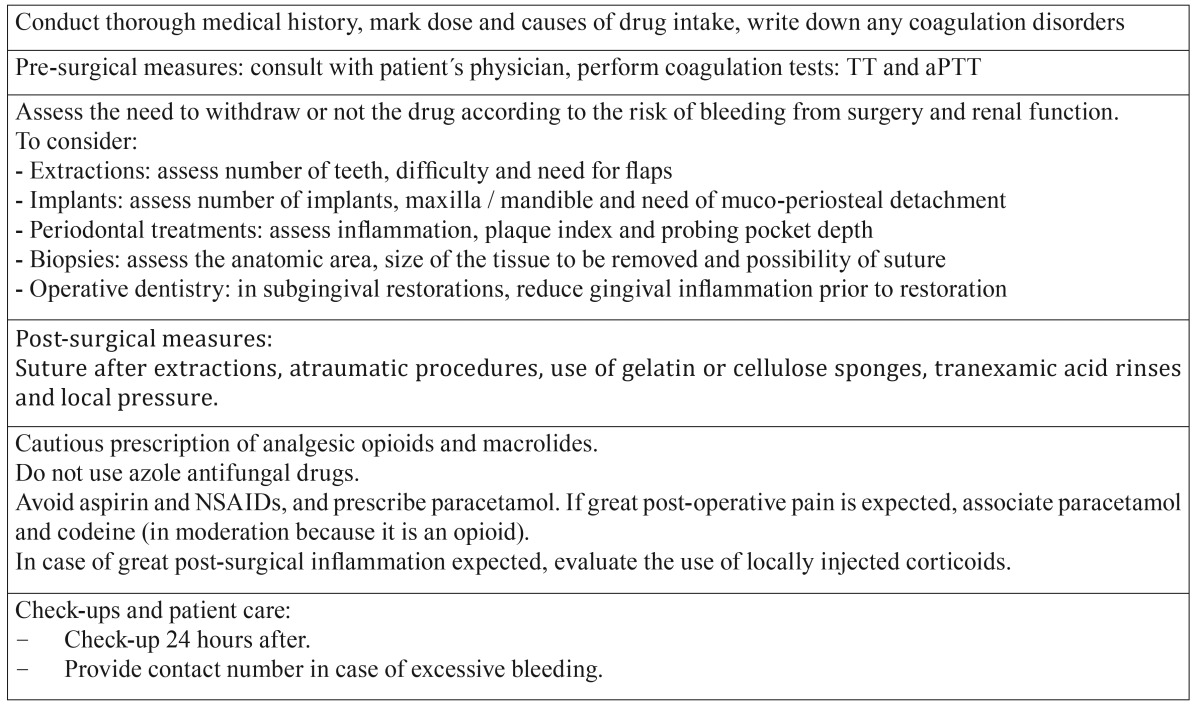
Dental recommendations when dealing with patients treated with dabigatran.

Always perform a thorough medical history, in which both the dose and the cause of drug intake are noted. It must include diseases related to coagulation disorders, such as liver disease. Before any procedure in which we anticipate bleeding, one should consult with the patient´s physician to develop a joint plan of action. This plan could include coagulation testing; it seems that the most appropriate and accessible tests in the case of dabigatran are TT and aPTT.

To decide whether or not to withdraw the drug before surgery, one must assess the risk of bleeding from the dental treatment and the patient´s renal function. Depending on the type of treatment to be performed, the practitioner must consider certain variables to establish the risk of bleeding. When extracting teeth, one will have to assess the number of teeth, the difficulty of the extractions, and the need for flaps. In case of implants, the number of implants to be placed will be assessed, if they are going to be placed in the maxilla or mandible (as the risk of bleeding will be different), and the need for muco-periosteal detachment. In periodontal therapy one should assess gingival inflammation, plaque index (PI) and pocket probing depth (PPD). If it is necessary to biopsy soft tissue, one should consider the anatomical area from which the sample is going to be taken, the size of the tissue to be removed, and whether or not suturing the area. As for restorative dentistry, if necessary to perform any subgingival restoration, it would be advisable to reduce gingival inflammation prior to restoration to lessen the risk of bleeding.

As discussed in other papers, it is necessary to perform a procedure as atraumatic as possible and after surgery, local measures such as the use of sutures, gelatin or cellulose sponges, tranexamic acid rinses and local pressure should be applied.

As for prescription medication, one should be cautious with opioid analgesics and macrolides; and azole antifungals are contraindicated. Overall, one should prescribe paracetamol as analgesic, and should avoid aspirin and NSAIDs; therefore, if post-surgical inflammation is anticipated, the use of locally injectable steroids should be assessed; and if great post-operative pain is expected, acetaminophen associated with codeine can be prescribed, always in moderation, since it is an opioid analgesic.

We believe appropriate to check on the patient within 24 hours of the surgical procedure, and always provide a contact number in case excessive bleeding occurs, as well as a referral to an emergency hospital to attend if necessary.

In conclusion, dabigatran is a drug that is a viable alternative to warfarin in the prophylactic treatment of stroke and systemic embolism in patients with atrial fibrillation. Therefore, we must take this into account when performing surgical and periodontal procedures to avoid the risk of postoperative bleeding, as well as, assess potential drug interactions with other drugs commonly used in dentistry. Although there are no well-designed clinical studies yet, existing studies agree to treat each patient taking dabigatran in an individual manner. It will be important to consider the risk of embolism and postoperative bleeding, and renal function of the patient. All procedures should be as minimally invasive as possible and appropriate local anti-hemolytic measures should be taken. However, more properly designed studies should be designed to determine a common protocol for treating these patients, just as we have for patients treated with warfarin or acenocoumarol.
